# Modern Water Treatment Technology Based on Industry 4.0

**DOI:** 10.3390/s25061925

**Published:** 2025-03-20

**Authors:** David Guth, David Herák

**Affiliations:** Department of Mechanical Engineering, Faculty of Engineering, Czech University of Life Sciences Prague, 165 00 Prague, Czech Republic; herak@tf.czu.cz

**Keywords:** autonomous water treatment, Industry 4.0, water quality, decentralized solutions, smart sensors

## Abstract

Access to clean water remains a critical global challenge, particularly in under-resourced regions. This study introduces an autonomous water treatment system leveraging Industry 4.0 technologies, including advanced smart sensors, real-time monitoring, and automation. The system employs a multi-stage filtration process—mechanical, chemical, and UV sterilization—to treat water with varying contamination levels. Smart sensors play a pivotal role in ensuring precise control and adaptability across the entire process. Experimental validation was conducted on three water types: pond, river, and artificially contaminated water. Results revealed significant reductions in key contaminants such as PPM, pH, and electrical conductivity, achieving water quality standards set by the WHO. Statistical analyses confirmed the system’s reliability and adaptability under diverse conditions. These findings underscore the potential of smart, sensor-integrated, decentralized water treatment systems to effectively address global water security challenges. Future research could focus on scalability, renewable energy integration, and long-term operational durability to enhance applicability in remote areas.

## 1. Introduction

Water plays a pivotal role in fostering prosperity by fulfilling fundamental human necessities, bolstering public health, sustaining livelihoods, driving economic growth, ensuring food and energy security, and safeguarding environmental integrity [1]. Despite its critical importance, the condition of surface and groundwater resources worldwide is declining due to a range of human activities, including industrial pollution, agricultural runoff, and urbanization [2,3]. Recent studies highlight that the integration of renewable energy sources into water treatment processes significantly improves efficiency while reducing environmental impact [4]. This is particularly problematic in developing countries, where many communities lack access to safe drinking water and must rely on alternative, often contaminated, sources such as wells, springs, and surface water reservoirs [5]. These communities are disproportionately affected by waterborne diseases and other health risks associated with poor water quality, further exacerbating issues of poverty and inequality [6].

Global challenges such as climate change, geopolitical instability, pandemics, mass migration, and economic crises like hyperinflation further deepen the disparities in water access [1]. Vulnerable and marginalized populations bear the brunt of these crises, facing elevated risks to their health and livelihoods. Rapid population growth, currently estimated at 80 million people per year, continues to drive increasing demand for freshwater resources, further straining already overburdened water supply systems [3]. In response to these pressures, innovative treatment technologies, including advanced membrane filtration and adsorption methods, have been developed to improve water quality and availability [7]. This rising demand is not matched by adequate improvements in water treatment infrastructure, particularly in regions where resources are scarce, and central water treatment facilities are non-existent [8].

In addition to the quantitative scarcity of water, the buildup of untreated wastewater exacerbates environmental and public health risks. Advanced oxidation processes (AOPs) have emerged as a key solution for removing persistent organic pollutants from water, offering a complementary approach to conventional methods [9]. Untreated wastewater contributes to the spread of infectious diseases, ecological phenomena such as eutrophication and water contamination, and the emission of greenhouse gases (GHGs), which further degrade air and water quality [6,10]. The challenge of providing clean, safe drinking water extends beyond merely increasing the quantity of water available; it also necessitates significant improvements in the quality of water being delivered, especially in areas where pollution levels are high and conventional treatment methods are insufficient [11].

Traditional water treatment technologies, such as coagulation, flocculation, sedimentation, filtration, and chemical disinfection, while effective in many urban settings, often fall short in handling the rapid fluctuations in water quality that are common in developing countries and remote areas [3]. These fluctuations are often driven by extreme weather events, seasonal variations, and industrial activities, which make water treatment an unpredictable and resource-intensive process [12]. The application of machine learning algorithms in monitoring and optimizing water treatment plants has shown great potential in predicting system failures and enhancing performance [13]. Moreover, the costs and technical expertise required to maintain these systems are prohibitive for many low-income regions, creating a pressing need for decentralized, low-maintenance, and affordable solutions that can adapt to varying water conditions in real-time [10]. Innovations in electrochemical water treatment technologies are addressing the challenges posed by high salinity levels in arid regions, proving effective in ensuring potable water availability [14].

The advent of Industry 4.0 technologies presents a groundbreaking opportunity to revolutionize water treatment systems by incorporating automation, real-time monitoring, and smart sensors. Industry 4.0, characterized by cyber-physical systems, the Internet of Things (IoT), big data analytics, and artificial intelligence, has already begun transforming industries such as manufacturing and energy management, and its application in water treatment holds immense potential [15]. Integrating these advanced technologies into water treatment processes can make systems more efficient, adaptable, and sustainable. Automated systems with real-time monitoring capabilities allow for more precise control over water quality parameters, such as pH, temperature, and contaminant levels, reducing the risk of human error and minimizing operational costs [3]. This is particularly valuable in remote or resource-constrained areas where skilled labor is scarce, and conventional systems are often impractical [10].

The specific aim of this study is to develop and validate an autonomous water treatment system capable of operating without human intervention. This system will integrate Industry 4.0 technologies—such as smart sensors and automated filtration processes—to ensure real-time water quality monitoring and control. The study will also assess the system’s effectiveness in consistently producing potable water that meets the stringent standards set by the World Health Organization (WHO), even in regions with highly variable water quality. While the article incorporates practical elements often seen in laboratory work for students practicing water cleaning techniques, its primary focus is on presenting a comprehensive scientific investigation into these methods. The content is supported by empirical data and rigorous analysis, aligning with the parameters of a scientific article.

## 2. Materials and Methods

The design and construction of the autonomous water treatment system were developed to ensure full automation and minimal human intervention. The following sections describe the components, configuration, and operation of the system in detail, allowing for the replication of the setup for similar water treatment applications.

### 2.1. System Design and Setup

**Figure 1:**
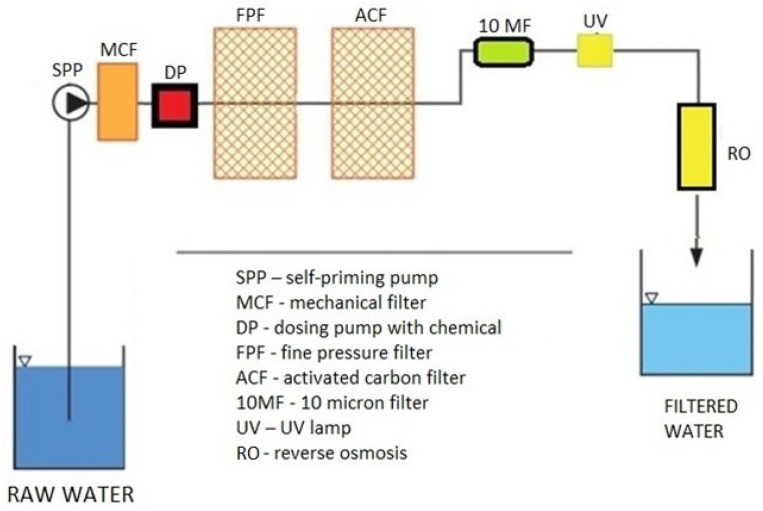
Diagram of the Water Treatment Plant.

The water treatment system is designed to handle various levels of water contamination and consists of multiple filtration stages, each aimed at removing specific types of pollutants. Figure 1 describes the individual filtration steps through which water is fed into the treatment plant. The system can be fully controlled remotely via a cloud-based interface that monitors real-time water quality parameters, including pH, temperature, and contaminant levels.

1. Water Source and Initial Intake:

The system is connected to a 1000 L untreated water tank, which simulates contaminated water sources such as ponds, rivers, or artificially contaminated samples. A self-priming pump with a press controller initiates the water intake, ensuring the optimal pressure for the filtration process. The press controller prevents pressure fluctuations, maintaining system stability and protecting downstream components.

Pump Specification:

- Model: XYZ Self-Priming Pump with Press controller;
- Operating pressure: 2–5 bar;
- Max flow rate: 50 L/min;
- Pressure threshold: Adjustable between 1 and 5 bar.

2. Mechanical Filtration (Cintropur NW500 Filter):

The first filtration stage involves a Cintropur NW500 mechanical filter equipped with a 10-micron cartridge. This filter is responsible for removing large particles such as sand, silt, and clay from the water before it enters more sensitive filtration stages.

Filter Specification:

- Cartridge size: 10 microns (replaceable);
- Maximum flow rate: 18 m^3^/h;
- Maximum operating pressure: 16 bar;
- Standard sleeve size: 25 microns;
- Function: Prevent damage to sensitive components in subsequent stages.

3. Chlorine Dosing (IWAKI EW F Electromagnetic Pump):

The system includes a chlorine dosing pump to ensure the chemical disinfection of the water. The dosing process is controlled by flow sensors, which adjust the amount of chlorine-based on real-time water flow measurements. The system is designed to ensure precise dosing to avoid under- or overdosing.

Pump Specification:

- Model: IWAKI EW F Electromagnetic Membrane Pump;
- Accuracy: ±1% dosing precision;
- Flow rate: Adjustable (0–10 L/h);
- Control mechanism: Sensor-based real-time adjustment via cloud monitoring.

4. Sand Filtration:

Following chemical dosing, the water undergoes sand filtration where it passes through a bed of sand to remove suspended solids. The sand filter features automatic piston heads for self-cleaning, which can be programmed based on contamination levels. The system’s software controls the cleaning cycles to optimize filtration without manual intervention.

Filter Specification:

- Sand grain size: 0.5–1.0 mm;
- Cleaning cycles: Programmable (daily or weekly based on contamination);
- Max flow rate: 20 m^3^/h;
- Piston heads: Automatic, with software-controlled cycles.

5. Activated Carbon Filtration (Silver-Enhanced):

The next stage uses an activated carbon filter, enriched with silver particles to enhance antimicrobial properties. This filter removes chlorine residuals, organic pollutants, and odors from the water.

Filter Specification:

- Carbon: Activated, silver-enhanced;
- Removal: Chlorine, organic pollutants, odors;
- Flow rate: 12 m^3^/h;
- Filter lifespan: Approximately 6–12 months depending on water quality.

6. UV Sterilization (Cintropur UV Lamp 2100):

The water then passes through a UV sterilization unit to destroy microbial contaminants. The Cintropur UV Lamp 2100 emits UV-C light at 254 nm, which is highly effective in eliminating bacteria, viruses, and other pathogens.

UV Lamp Specification:

- UV-C wavelength: 254 nm;
- Max flow rate: 2000 L/h;
- Lifespan: 9000 h;
- Maintenance: Lamp replacement after 9000 h of operation.

7. Reverse Osmosis (1600 GPD Unit):

The final stage of the filtration system is reverse osmosis (RO), which removes dissolved solids, heavy metals, and pathogens. The 1600 GPD reverse osmosis unit operates with a five-stage pre-filtration system to ensure maximum water purity.

RO Unit Specification:

- Capacity: 1600 gallons per day.
- Pre-filtration stages:
  1. 5-micron polypropylene filter (removes large debris);
  2. Activated carbon filter (removes chlorine and organic compounds);
  3. 1-micron polypropylene filter (removes finer debris);
  4. Reverse osmosis membrane (removes dissolved solids and heavy metals);
  5. Post-filtration activated carbon filter (enhances taste);
  6. Efficiency: 99% removal of dissolved solids.

### 2.2. Real-Time Monitoring and Control

The entire system is equipped with cloud-based monitoring software, allowing for real-time observation of key parameters such as pH, temperature, electrical conductivity (EC), and flow rate. The system includes a mobile application that provides remote access to the data, enabling operators to make adjustments without needing to be on-site. Toucan in the Elite program is a cloud monitoring system that was chosen for remote unit security. Using the control unit installed in the reverse osmosis, all values (membrane fouling, flow, pressure, amount of bacteria in the water, temperature and pH) were sent to the computer, where they were subsequently analyzed.

Data Collection Instruments:

- pH Meter: Combined pH P700 Pro 2;
- EC Meter: EC 4500 with automatic compensation;
- Temperature: Built-in probes for continuous monitoring;
- Cloud Connectivity: Secure data transmission to the operator’s mobile device.

### 2.3. System Validation and Testing

The system was tested under three different contamination scenarios: stagnant water (pond), flowing water (river), and artificially contaminated water. Coliform bacteria have been added to the contaminated water. Clear and serious fecal contamination in drinking water is, therefore, identified based on the determination of the coliform bacterium Escherichia coli, which is a reliable indicator of fecal contamination. Testing and analysis were conducted at VZ LAB, a certified water quality laboratory, to ensure accurate and reliable results.

Each scenario was tested over three weeks, with weekly measurements to capture any variations in filtration effectiveness and to ensure statistical reliability. This approach allowed for a detailed evaluation of the system’s performance over time and provided data for calculating mean values and standard deviations for each parameter.

### 2.4. Measurement Procedures and Instruments:

1. Electrical Conductivity (EC):

Instrument: Conductivity Meter, Model EC 4500 with automatic temperature compensation.

Procedure: The conductivity meter was calibrated before each set of measurements using a standard calibration solution (1413 µS/cm at 25 °C). For each sample, the probe was immersed in the water until the reading stabilized, and the measurement was recorded. Conductivity readings were taken both before and after filtration.

2. pH Measurement:

Instrument: pH Meter, Model pH P700 Pro 2.

Procedure: The pH meter was calibrated daily with standard buffer solutions (pH 4, 7, and 10) to ensure accuracy. Samples were collected in clean, non-reactive containers, and the pH probe was inserted and allowed to stabilize. The pH was measured before and after filtration to observe any changes resulting from the treatment process.

3. Parts Per Million (PPM):

Instrument: Total Dissolved Solids (TDS) Meter, calibrated to read in ppm.

Procedure: The TDS meter was used to measure the concentration of dissolved solids. Calibration was conducted using a TDS calibration solution. The probe was placed in the water sample, and readings were recorded once stable. Measurements were taken before and after filtration to determine the system’s effectiveness in reducing dissolved solid concentrations.

4. Conductivity Factor (CF):

Instrument: The CF was calculated as a derived value based on the EC readings using standardized conversion factors.

Procedure: CF was calculated for each sample by taking the EC reading and applying a conversion factor suited for the specific water type, as recommended by standard water analysis protocols. CF values were recorded before and after filtration.

5. Temperature:

Instrument: Built-in temperature probes in both EC and pH meters.

Procedure: Temperature was measured concurrently with EC and pH to ensure that readings were temperature-compensated. Data were recorded to assess any temperature variations that might affect water quality during filtration.

### 2.5. Data Collection and Analysis

Each test was repeated for three consecutive weeks, with measurements taken both before and after each filtration process. This repetition ensured the statistical reliability of the data. Data analysis included calculating mean values and standard deviations for each parameter across the three weeks.

A paired *t*-test was used to assess the statistical significance of differences in EC, CF, pH, and PPM before and after filtration, with results confirming the effectiveness of the treatment system (*p* < 0.05).

## 3. Results

**Figure 2:**
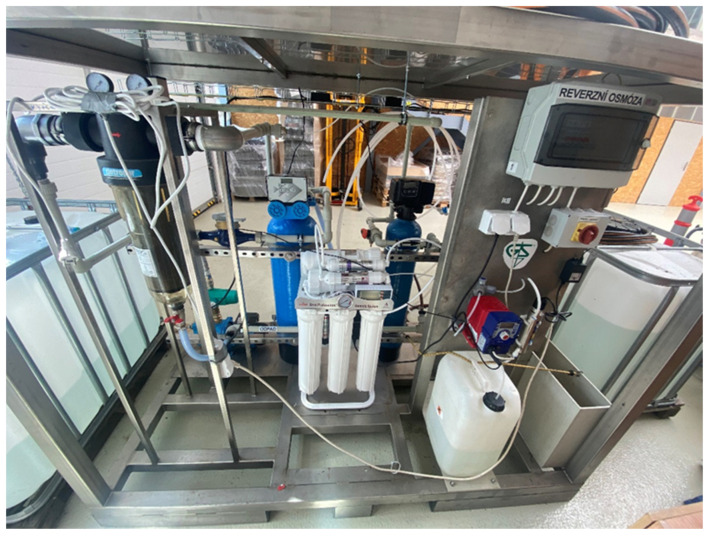
The Completed Experimental Unit.

The performance of the autonomous water treatment system was evaluated by measuring essential water quality parameters—Electrical Conductivity (EC), Conductivity Factor (CF), pH, Parts Per Million (PPM), and Temperature—before and after filtration. The study covered three water types: pond water, river water, and artificially contaminated water. Each experiment was conducted in triplicate to ensure statistical reliability. Statistical analyses, including mean values, standard deviations, 95% confidence intervals (CI), and paired *t*-tests (with a significance level of *p* < 0.05), were used to rigorously assess the filtration system’s effectiveness. Figure 2 demonstrates the final phase of the water treatment plant that has been built and is currently being tested.

### 3.1. Pond Water Testing

#### 3.1.1. Initial Water Quality (Before Filtration)

The initial pond water sample had high turbidity and an elevated pH, reflecting substantial contamination. The pre-filtration baseline values were:

- PPM: 203 ± 5 mg/L (95% CI: 198.34–210.26);
- pH: 9.7 ± 0.2 (95% CI: 9.60–9.80);
  ○ Substances such as sodium hydroxide (NaOH) or potassium hydroxide (KOH) are strong bases that can increase the pH of the solution. These substances were contained in the tested water. The alkaline environment is, therefore, caused by the content of hydroxide ions.
- EC: 0.21 ± 0.01 mS/cm (95% CI: 0.19–0.22);
- CF: 2.93 ± 0.15 (95% CI: 2.75–3.10);
- Temperature: 19.73 ± 0.31 °C (95% CI: 19.34–20.12).

#### 3.1.2. Post-Treatment Water Quality (After Filtration)

Following filtration, significant reductions were observed in several key parameters:

- PPM: 167.67 ± 3.06 mg/L (95% CI: 164.52–170.81);
- pH: 6.57 ± 0.15 (95% CI: 6.42–6.72);
- EC: 0.20 ± 0.0 mS/cm (95% CI: 0.20–0.20);
- CF: 2.30 ± 0.10 (95% CI: 2.20–2.40);
- Temperature: 20.40 ± 0.26 °C (95% CI: 20.10–20.69).

#### 3.1.3. Statistical Significance

The reduction in PPM and pH was statistically significant (*p* < 0.01), supporting the system’s efficacy in removing suspended solids and achieving acid-neutralization. Minor but statistically significant increases in temperature (*p* < 0.05) are attributed to system operation mechanics.

**Figure 3:**
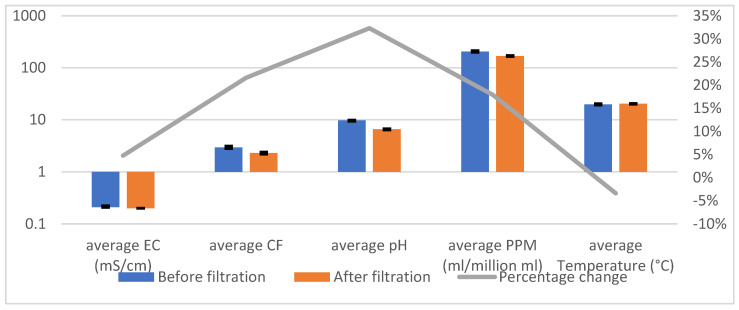
Pond Water Testing Graph.

Table 1 and Figure 3 illustrate the system’s effectiveness in treating pond water, demonstrating consistent reductions in key contaminants and significant pH optimization across three trials. The error bars in Figure 3 reflect the variability of the measurements (standard deviations), providing a clear indication of the system’s reliability. While the percentage change per parameter is still depicted, the emphasis is placed on the substantial improvements in water quality metrics, underscoring the system’s ability to achieve reproducible and effective filtration.

### 3.2. River Water Testing

#### 3.2.1. Initial Water Quality (Before Filtration)

The river water sample contained high levels of dissolved solids and organic contaminants, as shown by the following pre-filtration values:

- PPM: 911.33 ± 35.79 mg/L (95% CI: 868.26-954.41);
- pH: 8.23 ± 0.35 (95% CI: 7.79–8.67);
- EC: 1.0 ± 0.1 mS/cm (95% CI: 0.87–1.13);
- CF: 10.16 ± 0.26 (95% CI: 9.84–10.49);
- Temperature: 11.8 ± 0.4 °C (95% CI: 11.31–12.29).

#### 3.2.2. Post-Treatment Water Quality (After Filtration)

After filtration, substantial decreases were observed in key parameters:

- PPM: 183.0 ± 7.94 mg/L (95% CI: 173.77–192.23);
- pH: 6.92 ± 0.17 (95% CI: 6.68–7.16);
- EC: 0.29 ± 0.035 mS/cm (95% CI: 0.24–0.33);
- CF: 2.42 ± 0.10 (95% CI: 2.30–2.54);
- Temperature: 16.6 ± 0.43 °C (95% CI: 16.10–17.09).

#### 3.2.3. Statistical Significance

The observed reductions in PPM, EC, and CF were statistically significant (*p* < 0.01), supporting the system’s effectiveness in removing contaminants and improving water quality. The increase in temperature (*p* < 0.01) was expected and falls within operational norms.

**Figure 4:**
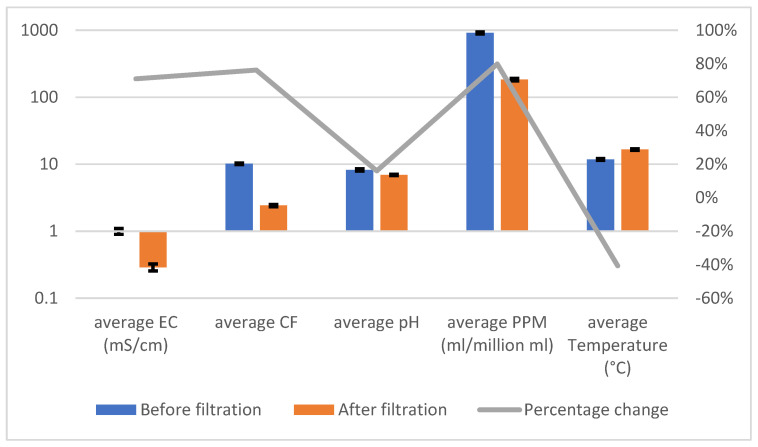
River Water Testing Graph.

Table 2 and Figure 4 demonstrate the system’s consistent effectiveness in treating river water, achieving significant reductions in PPM and CF across all trials. The error bars in Figure 4 highlight the variability in the measurements (standard deviations), providing additional insight into the system’s reliability. While the percentage decreases across parameters are visually represented, the emphasis remains on the substantial improvements in water quality metrics, particularly the system’s ability to effectively handle water with high levels of dissolved solids and contaminants.

### 3.3. Artificially Contaminated Water Testing

#### 3.3.1. Initial Water Quality (Before Filtration)

The artificially contaminated water sample posed high levels of chemical and organic pollutants:

- PPM: 600 ± 10 mg/L (95% CI: 586.6–613.4);
- pH: 5.5 ± 0.1 (95% CI: 5.37–5.63);
- EC: 0.59 ± 0.04 mS/cm (95% CI: 0.52–0.65);
- CF: 7.4 ± 0.10 (95% CI: 7.25–7.55);
- Temperature: 18.63 ± 0.15 °C (95% CI: 18.42–18.84).

#### 3.3.2. Post-Treatment Water Quality (After Filtration)

The filtration process achieved substantial improvements:

- PPM: 171.33 ± 7.02 mg/L (95% CI: 162.81–179.86);
- pH: 6.7 ± 0.05 (95% CI: 6.63–6.77);
- EC: 0.29 ± 0.01 mS/cm (95% CI: 0.27–0.30);
- CF: 2.35 ± 0.05 (95% CI: 2.28–2.42);
- Temperature: 20.13 ± 0.26 °C (95% CI: 19.84–20.42).

#### 3.3.3. Statistical Significance

The reductions across PPM, pH, EC, and CF were statistically significant (*p* < 0.01), highlighting the system’s robust capability in managing high pollution loads. The slight increase in temperature (*p* < 0.05) aligns with operational expectations.

**Figure 5:**
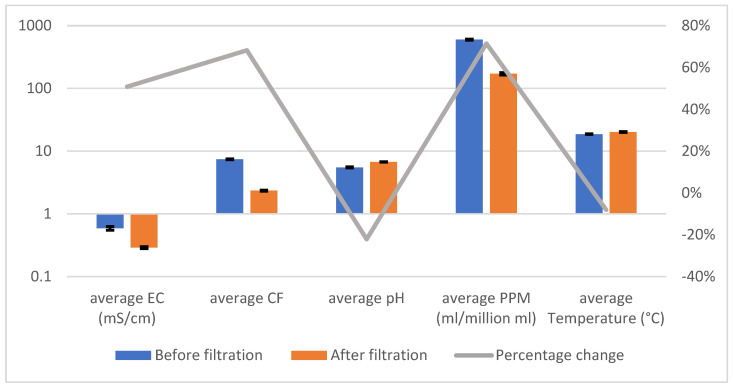
Artificially Contaminated Water Testing Graph.

Table 3 and Figure 5 confirm the filtration system’s effectiveness in addressing chemically and organically complex contaminants, achieving consistent reductions in PPM and CF across all trials. The error bars in Figure 5 illustrate the variability in the results (standard deviations), further demonstrating the system’s reliability under challenging conditions. While percentage changes remain highlighted, the focus is on the system’s capacity to adapt and effectively treat heavily polluted water.

### 3.4. Summary of Statistical Reliability

Each experiment was repeated three times, and the narrow 95% confidence intervals and low standard deviations demonstrate the system’s robust and consistent performance. The statistically significant changes across PPM, pH, EC, and CF for all water types (*p* < 0.01) confirm that improvements were achieved through the filtration system, rather than random variation. This reliability indicates potential applicability for deployment in various water treatment scenarios, especially in regions with high contamination needs and limited resources.

During conducted research, emphasis was placed on maximizing the automation of the water treatment unit and ensuring a rapid response to fluctuations in incoming water quality. A self-sufficient unit was developed using sensors and Industry 4.0 technologies, enabling remote operation, real-time data collection, and analysis. In the event of hazardous substance detection, an immediate shutdown can be initiated, pre-venting significant damage and ensuring operational safety.

## 4. Discussion

This study demonstrates the effectiveness of an autonomous water treatment system across three contamination scenarios, emphasizing its ability to significantly reduce contaminants in pond, river, and artificially contaminated water. The multi-stage filtration design, combining mechanical and chemical processes with real-time monitoring, demonstrates strong potential for practical applications, particularly in regions facing severe contamination challenges or limited water infrastructure. In this section, the findings are critically analyzed in the context of recent advancements in water treatment technologies, with a focus on their broader implications for global water security. In follow-up research, we focus on harmful substances contaminating water, especially microplastics, which can have serious impacts on human health.

### 4.1. Scientific Contribution and System Efficiency

The key scientific contribution of this study lies in the integration of autonomous technologies within water treatment, enabling high operational efficiency with minimal manual intervention. The significant reductions in PPM, pH, CF, and EC across diverse water types underscore the system’s adaptability and robustness. Compared to conventional systems that require frequent maintenance and manual operation [5,10], the autonomous design presented here minimizes operational complexity while ensuring consistent improvements in water quality. Similar findings were reported in the study [16], who demonstrated the potential of autonomous systems in reducing operational costs and improving adaptability in water treatment.

The system’s real-time monitoring and adaptive capabilities stand out as critical innovations, addressing a growing demand for water treatment solutions that can respond dynamically to fluctuating contamination levels. Similar advancements have been highlighted in studies [14,16], which emphasized the importance of integrating sensor-based optimization into filtration systems to enhance their performance in variable water quality conditions. Recent advancements in IoT-based water monitoring have further enhanced the precision of real-time adjustments, as explored in the study [17]. Notably, the system evaluated here demonstrates consistent reductions in pH for highly alkaline water, stabilizing it within WHO standards for drinking water—a challenge often unmet by single-stage filtration technologies [1,12].

The observed stability of EC in treated pond water highlights the system’s ability to preserve ionic balance—a key factor in ensuring water suitability for local ecosystems and agricultural applications. This finding aligns with [18], who emphasized the ecological importance of maintaining mineral content in treated water. Furthermore, the system’s automatic cleaning cycles mitigate common issues such as clogging—a limitation identified in conventional filtration systems operating under high turbidity conditions [17]. The study [19] also identified cleaning mechanisms as a vital component for enhancing the longevity and efficiency of filtration systems in high-turbidity environments.

### 4.2. Comparison with Existing Water Treatment Technologies

The results build on existing advancements in multi-stage filtration technologies, offering a comprehensive approach to reducing both organic and inorganic pollutants. Traditional systems often rely on specific methods, such as activated carbon for organic compounds or ion exchange for heavy metals [2,12] whereas the system presented here achieves simultaneous reductions across multiple parameters. This versatility is particularly significant in treating water with high contamination variability, where single-stage systems often fail to adapt effectively [20]. Recent innovations in advanced membrane technologies have further expanded the range of treatable contaminants, as demonstrated by the study [21].

The system’s reduction in PPM and CF, for instance, supports findings in the study [11], who demonstrated the efficiency of multi-stage filtration in reducing particulate matter in pesticide-contaminated water. However, this study expands upon those findings by incorporating automation and adaptive monitoring, ensuring continuous operation even under challenging conditions. The ability to reduce pH from extreme alkalinity without the need for external buffering agents also represents a substantial improvement over many conventional systems, as previously noted in the study [3]. This aligns with research by the study [22], which highlighted the limitations of conventional systems in handling pH stabilization in highly alkaline environments.

The technology used in water treatment is at a high level of maturity, but current trends and technological progress require continuous improvement of water quality and optimization of the treatment processes. Modern water treatment plants are now equipped with systems for automatic measurement of water hardness and its subsequent treatment in real time to meet the requirements set by the World Health Organization (WHO). A control system connected to a flow meter monitors water quality and, in case of detection of increased hardness, applies antiscalant before the pre-filtration process in the reverse osmosis unit.

Compared to more advanced and more expensive systems, some treatment plants like ECO Soft technology, which allows recycling of part of the concentrate back into the pump, thus optimizing water use and minimizing waste are mentioned in Table 4. The Ecosoft RO system operates fully automatically, and when the clean water tank is filled, it stops automatically. The membrane rinsing process is programmed for several modes—it always takes place before switching to standby mode, at predefined intervals during standby mode and during equipment maintenance.

### 4.3. Significance for Current Global Challenges

Globally, access to clean water is increasingly threatened by rising levels of pollutants, including pharmaceutical residues, microplastics, and heavy metals, which are now pervasive in both urban and rural water supplies [23]. This study directly addresses these challenges by demonstrating a filtration system capable of treating a wide range of contaminants. Unlike conventional systems designed for stable and predictable contamination levels, the adaptive nature of this system makes it particularly suited for regions affected by climate change, where water quality is highly variable [16].

The importance of such innovations cannot be overstated. According to [8], nearly 2 billion people globally lack access to safely managed drinking water, with rural communities disproportionately affected. This system’s autonomous operation, requiring minimal manual intervention, aligns with global efforts to expand access to clean water in under-resourced regions. Furthermore, integrating renewable energy sources, such as solar power, with these systems could enhance their applicability in remote and off-grid areas, as demonstrated by the study [17].

In the broader context of Sustainable Development Goal 6 (SDG 6), which emphasizes universal access to clean water and sanitation, this system contributes directly by addressing both quality and accessibility challenges. By providing a reliable and efficient means of treating water in diverse conditions, the system holds promise for mitigating the impacts of water scarcity exacerbated by climate-driven contamination events [1].

### 4.4. Limitations and Areas for Future Research

While the system’s effectiveness has been demonstrated across controlled scenarios, further research is needed to evaluate its performance in addressing more complex and emerging contaminants. Future studies should prioritize:

1. Testing against contaminants of emerging concern (CECs);
  ○ These include pharmaceutical residues, endocrine disruptors, and microplastics, reflecting the increasing complexity of wastewater contaminants and the challenges in maintaining material stability under various environmental and operational conditions [24].
2. Long-term durability;
  ○ Investigating how the system performs under continuous operation over extended periods, particularly in regions with extreme environmental conditions.
3. Economic feasibility;
  ○ Conducting cost–benefit analyses to determine the system’s financial viability compared to existing technologies, especially in low-income regions.
4. Integration with renewable energy.
  ○ Exploring the potential for solar- or wind-powered operations to further reduce its environmental footprint.

### 4.5. Practical Implications and Broader Applications

The autonomous nature of this system addresses critical barriers to clean water access in under-resourced regions, where infrastructure and skilled labor are often lacking. Its real-time monitoring capabilities provide an added layer of reliability, allowing for remote adjustments to filtration settings in response to changing water quality. This adaptability is crucial in addressing the dynamic challenges posed by climate change, including flood-related contamination events and prolonged droughts [3,23].

Furthermore, the system’s modular design makes it suitable for integration into decentralized water treatment networks, which are increasingly seen as a viable alternative to centralized systems in regions with limited infrastructure. Its potential applications extend beyond drinking water treatment to include agricultural irrigation, industrial reuse, and emergency relief operations, demonstrating its broad utility [25].

## 5. Conclusions

This study highlights the effectiveness of a modern, autonomous water treatment technology rooted in Industry 4.0 principles. The integration of a multi-stage filtration system with advanced automation and remote monitoring capabilities positions this technology as a significant improvement over conventional water treatment systems, especially in addressing the diverse needs of both remote and under-resourced regions. The filtration system’s demonstrated ability to consistently reduce contaminant levels in varying water sources—including pond, river, and artificially contaminated water—underlines its robustness and adaptability to a wide range of water quality challenges.

The system’s low maintenance requirements and capacity for real-time, remote monitoring make it a practical solution for areas with limited access to skilled personnel and technical resources. By utilizing smart sensors, automated controls, and cloud-based data management, this technology maintains water quality standards in line with the WHO guidelines, providing a reliable and sustainable solution with minimal manual intervention. The system’s flexibility in adjusting rinsing cycles, tailored to pollutant levels from mild to high contamination, further supports its application across diverse environments.

Future research could expand on this study by investigating the system’s scalability and long-term durability under different environmental and seasonal conditions. Additionally, exploring its efficacy in filtering out specific contaminants such as heavy metals and pharmaceutical residues would deepen understanding of its broader applicability. Nonetheless, the current findings indicate that this autonomous water treatment technology is a highly effective, adaptable, and sustainable solution for ensuring clean and safe water, with the potential to meet the growing global demand for accessible drinking water across various urban and rural contexts.

## Figures and Tables

**Table 1:** Pond Water Testing.

	EC Before	EC After	CF Before	CF After	pH Before	pH After	PPM Before	PPM After	Temp. Before	Temp. After
W1	0.20	0.20	2.90	2.40	9.70	6.80	203.00	171.00	19.40	20.10
W2	0.22	0.20	3.10	2.20	9.80	6.50	210.00	168.00	19.80	20.50
W3	0.21	0.20	2.80	2.30	9.60	6.40	200.00	164.00	20.00	20.60

**Table 2:** River Water Testing.

	EC Before	EC After	CF Before	CF After	pH Before	pH After	PPM Before	PPM After	Temp. Before	Temp. After
W1	1.00	0.30	10.00	2.50	8.20	7.10	904.00	184.00	11.40	16.00
W2	1.10	0.25	10.50	2.30	8.60	6.80	950.00	190.00	11.80	17.00
W3	0.90	0.32	9.99	2.45	7.90	6.85	880.00	175.00	12.20	16.80

**Table 3:** Artificially Contaminated Water Testing.

	EC Before	EC After	CF Before	CF After	pH Before	pH After	PPM Before	PPM After	Temp. Before	Temp. After
W1	0.60	0.30	7.30	2.30	5.40	6.80	590.00	179.00	18.50	20.10
W2	0.55	0.28	7.50	2.40	5.60	6.70	610.00	165.00	18.60	19.90
W3	0.62	0.29	7.40	2.35	5.50	6.65	600.00	170.00	18.80	20.40

**Table 4:** Comparison of technical specifications.

	WATEX MO-2 Ecosoft	PURE FLOW 1600 GPD INDUSTRIAL
Flow capacity	2 m^3^/h	0.26 m^3^/h
Permeate recovery	75%	65%
Maximum TDS	3000 mg/L	2200 mg/L
Influent flow rate (service)	2.7–4 m^3^/h	0.4–0.6 m^3^/h
Influent flow rate (rinse)	10 m^3^/h	1.1 m^3^/h
Inlet pressure of water	2–4 bar	4–6 bar
Operating pressure	8–12 bar	10–12 bar
Electrical requirements	3 × 380 V, 50 Hz	220–380 V, 50/60 Hz
Electrical power	3 kW	2–3 kW

## Data Availability

The original contributions presented in the study are included in the article; further inquiries can be directed to the corresponding author.
